# Evaluating and optimizing Acid-pH and Direct Lysis RNA extraction for SARS-CoV-2 RNA detection in whole saliva

**DOI:** 10.1038/s41598-024-54183-w

**Published:** 2024-03-25

**Authors:** Brayden LaBute, Jackie Fong, Farinaz Ziaee, Robert Gombar, Mathew Stover, Terry Beaudin, Maria Badalova, Qiudi Geng, Ryland Corchis-Scott, Ana Podadera, Kyle Lago, ZhenHuan Xu, Fievel Lim, Felix Chiu, Minghua Fu, Xiaofeng Nie, Yuanmin Wu, Corrina Quan, Caroline Hamm, R. Michael McKay, Kenneth Ng, Lisa A. Porter, Yufeng Tong

**Affiliations:** 1 Department of Biomedical Sciences, University of Windsor, Windsor, ON Canada; 2 Department of Chemistry and Biochemistry, University of Windsor, Windsor, ON Canada; 3 WE-SPARK Health Institute, University of Windsor, Windsor, ON Canada; 4 Great Lakes Institute for Environmental Research, University of Windsor, Windsor, ON Canada; 5 Aumintec Research Inc., Richmond Hill, ON Canada; 6 Windsor Regional Hospital, Windsor, ON Canada

**Keywords:** Assay systems, Infectious diseases, PCR-based techniques

## Abstract

COVID-19 has been a global public health and economic challenge. Screening for the SARS-CoV-2 virus has been a key part of disease mitigation while the world continues to move forward, and lessons learned will benefit disease detection beyond COVID-19. Saliva specimen collection offers a less invasive, time- and cost-effective alternative to standard nasopharyngeal swabs. We optimized two different methods of saliva sample processing for RT-qPCR testing. Two methods were optimized to provide two cost-efficient ways to do testing for a minimum of four samples by pooling in a 2.0 mL tube and decrease the need for more highly trained personnel. Acid-pH-based RNA extraction method can be done without the need for expensive kits. Direct Lysis is a quick one-step reaction that can be applied quickly. Our optimized Acid-pH and Direct Lysis protocols are reliable and reproducible, detecting the beta-2 microglobulin (*B2M*) mRNA in saliva as an internal control from 97 to 96.7% of samples, respectively. The cycle threshold (Ct) values for *B2M* were significantly higher in the Direct Lysis protocol than in the Acid-pH protocol. The limit of detection for *N1* gene was higher in Direct Lysis at ≤ 5 copies/μL than Acid-pH. Saliva samples collected over the course of several days from two COVID-positive individuals demonstrated Ct values for N1 that were consistently higher from Direct Lysis compared to Acid-pH. Collectively, this work supports that each of these techniques can be used to screen for SARS-CoV-2 in saliva for a cost-effective screening platform.

## Introduction

As of September 2023, the COVID-19 pandemic was still a reality with the SARS-CoV-2 Omicron variant and subvariants circulating throughout both vaccinated and unvaccinated populations^1,2^. The virus continued to mutate and spread, all while many countries relaxed most, if not all, their COVID-19 public health measures. The need for effective testing methods was a key concern throughout the pandemic, and rapid detection methods were an important tool in the prevention of outbreaks by identifying positive individuals and removing them from the population. The speed and accuracy of detection are particularly important in protecting high-risk populations, such as residents of long-term care/retirement homes and immunocompromised individuals. In other areas, such as university campuses and amongst high-performance athletes (e.g. National Basketball Association, National Hockey League), rapid testing methods were used to effectively control the spread of COVID-19, preventing further death and long-term illness in communities^3–5^. Screening methods can also play an important role in preventing study or work disruptions, thereby protecting global economies. When combined with population-based surveillance, such as wastewater-based virus detection, rapid screening of individuals can be a powerful platform in protecting society from the devastating consequences of pandemics like COVID-19^6–8^.

Point-of-care rapid antigen tests were widely distributed throughout the latter half of the pandemic across Canada and were relied upon to test at home for COVID-19. Despite the advantages of convenience, availability and speed, these tests have a possibility of false negatives, either due to a low viral load at the time of the test or, more worrisome, a potentially reduced sensitivity to the newest variants^9,10^. Rapid antigen tests may give a false sense of security to individuals who exhibit symptoms of COVID-19 but test negative. PCR testing is more reliable for early detection of SARS-CoV-2 than antigen testing^11^. The primary mode of acquiring specimens from prospective cases within the public health system in Canada was nasopharyngeal swabs (NPS), and even at this time, this remains the current gold standard for COVID-19 PCR testing. Using NPS is costly and impractical when considering mass testing because NPS is invasive and needs a properly trained worker to be present and be in close contact to perform the test^4,12^. One potential way to test rapidly by PCR while decreasing cost and increasing participant compliance is using saliva and integrating the pooling of samples^13^.

Saliva can contain viral nucleic acid fragments shed by an infected individual, which can be utilized to predict and diagnose several infectious viral diseases, including cytomegalovirus, Zika virus, Epstein Barr virus, HIV, Human Papillomavirus (HPV) and Hepatitis A, B and C^14–20^. In the case of COVID-19, the salivary glands are a known reservoir for the SARS-CoV-2 virus^21^. Several studies have shown varying degrees of concordance between saliva and NPS testing, ranging from 87 to 100%, making it a suitable option^22–25^. As the virus has continued to mutate, the Omicron VOC has become more readily detectable with a higher viral load in saliva than previous variants^26,27^. Furthermore, saliva has many other benefits for testing for COVID-19. Saliva collection is non-invasive and can be performed without the need for a trained healthcare professional. Additionally, saliva has the advantage of ease of use for pooling samples^28,29^. In congregate living settings like dormitories in universities and colleges, or long-term care homes and shelters, pooled sampling can be valuable for detecting COVID-19 in groups. Pooled testing is necessary when disease prevalence is low to decrease the time and costs associated with testing^30,31^. Regular saliva PCR testing could be valuable in reducing the spread of lingering infections of COVID-19 as cases decline across populations. These advantages must be weighed when considering the gold standard of testing used for populations long-term.

Here we report the optimization of two RT-qPCR methods for detecting COVID-19 in saliva in a cost- and time-efficient manner. The rationale for optimizing two methods, the Acid-pH and Direct Lysis protocols, was to be able to pool a minimum of four saliva samples of ample volume and process them in a 2.0 mL tube to enable analysis using a standard microcentrifuge. Multiple methods of extraction from saliva samples were tested, with the goal of establishing a safe, efficient, and reliable method of screening students, faculty, and staff study volunteers within a university population.

## Materials and methods

### Ethics and approval

The research performed involved human participants and was approved by the Research Ethics Board at the University of Windsor under REB# 21-005: "Testing the Feasibility and Optimizing Processes of a UWindsor COVID-19 Molecular Surveillance Laboratory". All research was performed in accordance with relevant guidelines/regulations. Electronically signed informed consent was obtained from all study participants and questionnaire data were collected and stored with REDCap^32,33^.

### Saliva sample collection

Self-administrated saliva specimen collection was carried out by the participants in a designated collection site at the University of Windsor from 10 AM to 12 PM, and 1 PM to 4 PM from Tuesday to Thursday from March 2021 to September 2022. The participants of the study were recruited through campus advertisement and registered in a REDCap database and their identities were anonymized. They were informed not to eat or drink for at least 60 min and 30 min, respectively, before providing a saliva sample. Signs were posted at the collection site to direct the participants to follow instructions step-by-step. Before entering the collection room, the participants sanitized their hands with 70% ethanol spray and then selected a pre-labeled 2.0 mL screw cap microtube containing 25 µL of RNAsecure™ (ThermoFisher) solution. An individually wrapped 1.0 mL sterile transfer pipette (VWR) was used to retrieve saliva from their mouth to fill to the 500 µL mark of the microtube. After capping the tube, the participants sanitized their hands again and placed the microtube containing the specimen onto a heat block kept at 60 °C on site. Before leaving the room, the participants wiped down all surfaces using 70% ethanol spray to sanitize the collection desk. At the end of the day, all samples were heated for a minimum of 10 min at 60 °C (as per the manufacturer’s recommendation for the activation of RNASecure™), and then at 95 °C for 5 min to ensure the sample was fully heat-inactivated before transporting to the lab for processing.

### Concentrated Acid-pH RNA extraction from whole saliva samples

The fundamental basis for the extraction of RNA from whole saliva was first developed and reported by Wozniak et al. 2020 and the method coined “The Acid-pH Method”^34^. In the initial adaptation of this protocol, the lysis buffer was concentrated 3× to reduce the volume of buffer used. It was then renamed the “Concentrated Acid-pH Method” to distinguish it from the original iteration. To prepare the concentrated lysis buffer, the solution was incubated at 60 °C for 10 min to allow the SDS to dissolve into solution prior to adjusting the pH. For an individual saliva sample, 100 µL of the concentrated lysis buffer (208.2 mM SDS, 204 mM sodium citrate dihydrate, 396 mM citric acid anhydrous, 30 mM EDTA, pH = 5.0) was added to 200 µL of saliva and vortexed. Afterwards, 150 µL of precipitation buffer (17 mM sodium citrate dihydrate, 33.3 mM citric acid anhydrous, and 4 M NaCl) was added to the lysates and vortexed to mix. The sample was incubated on ice for 5 min prior to centrifugation at 15,000 RCF for 6 min at room temperature. The supernatant (450 µL) was transferred to a new 1.5 mL microcentrifuge tube containing 450 µL of isopropanol, vortexed, and incubated at room temperature for 10 min. The sample was centrifuged at 15,000 RCF for 6 min at room temperature and the supernatant discarded. Pellets were washed with 300 µL of ice-cold wash buffer (70% ethanol in DEPC-treated water) and centrifuged at 15,000 RCF for 6 min at room temperature. After centrifugation, the supernatant was discarded and then the pellets were briefly centrifuged once more to pool any remaining ethanol removed prior to air-drying with the caps open for at least 5 min. To resuspend the pellet, 45 µL of pre-warmed (60 °C) PCR-grade water was added and incubated in a 60 °C water bath for 10 min.

### Optimized Acid-pH method

The Acid-pH RNA extraction was further optimized as the research study proceeded. In the final optimized protocol, 200 μL of saliva was added to a tube with 100 μL lysis buffer (69.4 mM SDS, 68 mM Sodium Citrate dihydrate, 132 mM Anhydrous Citric Acid, 10 mM EDTA, pH = 5.0) and vortexed. Then 150 μL of precipitation buffer was added and the samples were incubated at − 20 °C for 5 min, followed by a spin for 6 min at 15,000 RCF. Optional: the supernatant was transferred to a new empty 1.5 mL Eppendorf tube and centrifuged for 3 min at 15,000 RCF to remove any residual precipitate. The supernatant was then added to a 1.5 mL microcentrifuge tube containing 450 μL of isopropanol. The mixture was vortexed and incubated for 10 min at room temperature. The samples were centrifuged for 6 min at 15,000 RCF and the isopropanol was removed. 300 μL of ice-cold 70% ethanol was added, and the samples were centrifuged at 15,000 RCF for 5 min. The supernatant was discarded followed by a pulse spin to pool any remaining ethanol. This was removed completely, and the RNA pellets were air-dried with the caps open for 3–5 min. To resuspend the pellet, 50 μL of pre-warmed DEPC water (60 °C) was added and the samples were incubated at 60 °C in a water bath for 5 min. RNA samples could then be used for PCR and stored long-term at − 80 °C.

### Direct Lysis method

For individual samples, 54 µL of saliva were added into a new 1.5 ml microcentrifuge tube with 6 µL of 10X lysis buffer (2.5% (v/v) IGEPAL® CA-630 (Sigma Aldrich), 20 mM sodium citrate, 100 mM Tris–HCl, pH = 7.4) and were then vortexed to mix. The lysate was then used for PCR analysis. Further optimization of Direct Lysis added a brief pulse centrifuge (up to 2000 RCF) before RT-qPCR.

### PCR amplification

The SARS-CoV-2 nucleocapsid (N1) gene was chosen as the target sequence to identify cases of COVID-19. To detect the presence of the N1 and the internal controls B2M and RNase P, a one-step RT-qPCR reaction was performed on Acid-pH RNA extracts or Direct Lysis samples. For PCR with RNase P internal control, 10 μL of RNA extract was added to each PCR reaction containing the following: 15 μL 2 × Takyon One-Step RT Probe MasterMix (Eurogentec), 0.9 μL N1 primer mix (10 μM), 0.3 μL N1 probe (10 μM), 0.25 μL RNase P primer/probe mix (Thermo Scientific), 1.2 μL MgCl_2_ (2 mM), and 2.3 μL PCR grade water. The initial PCR with B2M control was performed by adding 10 μL of RNA extract to each PCR reaction containing the following: 15 μL 2 × Takyon One-Step RT Probe MasterMix (Eurogentec), 0.9 μL N1 primer mix (10 μM), 0.3 μL N1 probe (10 μM), 0.15 μL B2M primer mix (10 μM), 0.15 μL B2M probe (10 μM), 1.2 μL MgCl_2_ (2 mM), and 2.3 μL PCR grade water. The optimized PCR with B2M internal control was performed as follows: 10 μL of extract was added to each PCR reaction containing the following: 15 μL 2 × Takyon One-Step RT Probe MasterMix (Eurogentec), 0.9 μl N1 primer mix (10 μM), 0.3 μl N1 probe (10 μM), 0.3 μl B2M primer mix (10 μM), 0.3 μl B2M probe (10 μM), 1.2 μl MgCl_2_ (2 mM), and 2.3 μl PCR grade water. The PCR protocol was performed on a MA-6000 Real-Time Quantitative Thermal Cycler (Suzhou Molarray Co. LTD.) as follows: 40 °C (10 min), 95 °C (5 min), [95 °C (15 s), 60 °C (50 s)] × 42 cycles. The primer and probe sequences for N1 were designed by the CDC. SARS-CoV-2 Viral-Like Particles (VLPs) (SeraCare) were used as the positive template control for PCR reactions. This was prepared by adding 2.5 μL of VLPs and 2.5 μL Direct lysis buffer to 20 μL of DEPC-treated water.

### Figures and statistics

**Figure 1:**
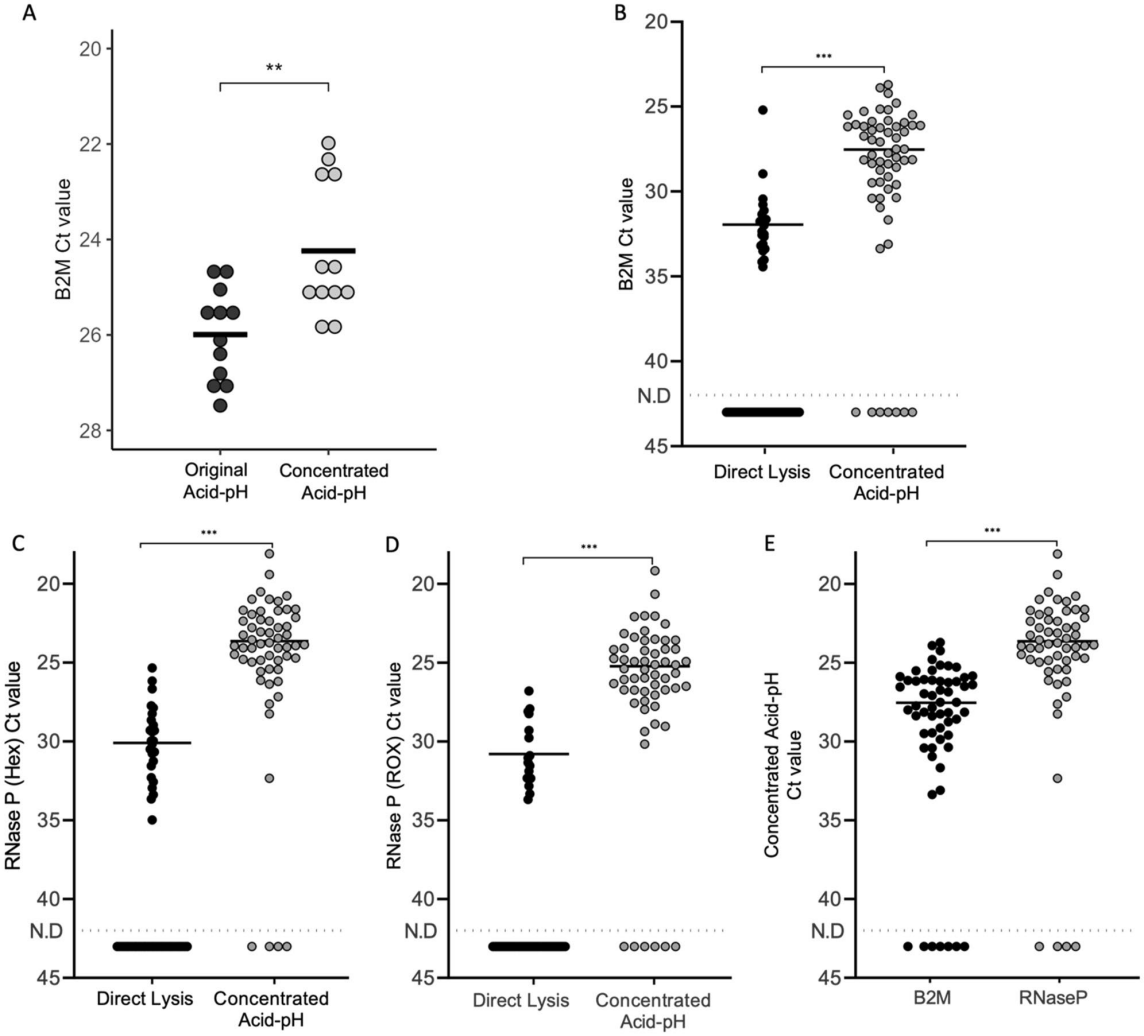
Testing the performance of concentrated Acid-pH and Direct Lysis Methods. The B2M Ct values of the original Acid-pH RNA extraction method was compared to the Concentrated Acid-pH method (with 3 × concentrated and less volume of lysis buffer) using whole saliva samples. n = 3 experiments, 2 samples per experiment, 2 technical replicates per sample. **Significant at p < 0.01, t-test. Average Ct values are denoted by black bars. Then, 58 saliva samples were used to test the efficacy of Concentrated Acid-pH and Direct Lysis Methods. This was tested for the detection of the internal control (**B**) B2M as well as RNase P in both the (**C**) HEX and (**D**) ROX channels. (**E**) Additionally, the detection of B2M and RNase P were compared to each other in Acid-pH RNA. ***Significant at p < 0.001, paired t-test. Average Ct values are denoted by black bars. The dashed line represents the detection limit where there was no detection (N.D).

The figures and statistics for Fig. 1B–E were generated using GraphPad Prism Ver. 8.0 software. The remaining figures and statistics were generated using R. The Shapiro–Wilk normality test was used to test for normal distribution in the data. Parametric or non-parametric testing was then performed based on whether the data was normally distributed or not respectively.

## Results

### Detection of B2M and RNase P internal controls between the Direct Lysis and Acid-pH methods

The processing of saliva samples came in the form of two different methods. The first method was based on the Acid-pH method developed by Wozniak and colleagues in 2020. This was a true RNA extraction method, with the added benefit of not using expensive kits. The method was altered slightly at the start of the study, and renamed the Concentrated Acid-pH method, because of the more concentrated lysis buffer concentration used that allowed for greater sample volume and pooling of samples in a 2.0 mL microcentrifuge tube. When we compared the original Acid-pH buffer to the Concentrated Acid-pH using whole saliva and analyzing for B2M, we found that the B2M Ct values for the Concentrated Acid-pH method were significantly lower than the original method (p < 0.01, Fig. 1A). The second extraction-free method used was termed “Direct Lysis”. This was a quick method developed by Aumintec Inc. Additionally, this method requires only 54 μL of saliva, in contrast to the 200 μL needed for Concentrated Acid-pH. To test the performance of the Concentrated Acid-pH and Direct Lysis protocols, saliva samples from 58 participants were collected and processed. RNase P and B2M internal controls were used to test the efficacy of these two extraction methods. These are both internal controls that are suitable for RT-qPCR with saliva extracts^34,35^.

**Figure 2:**
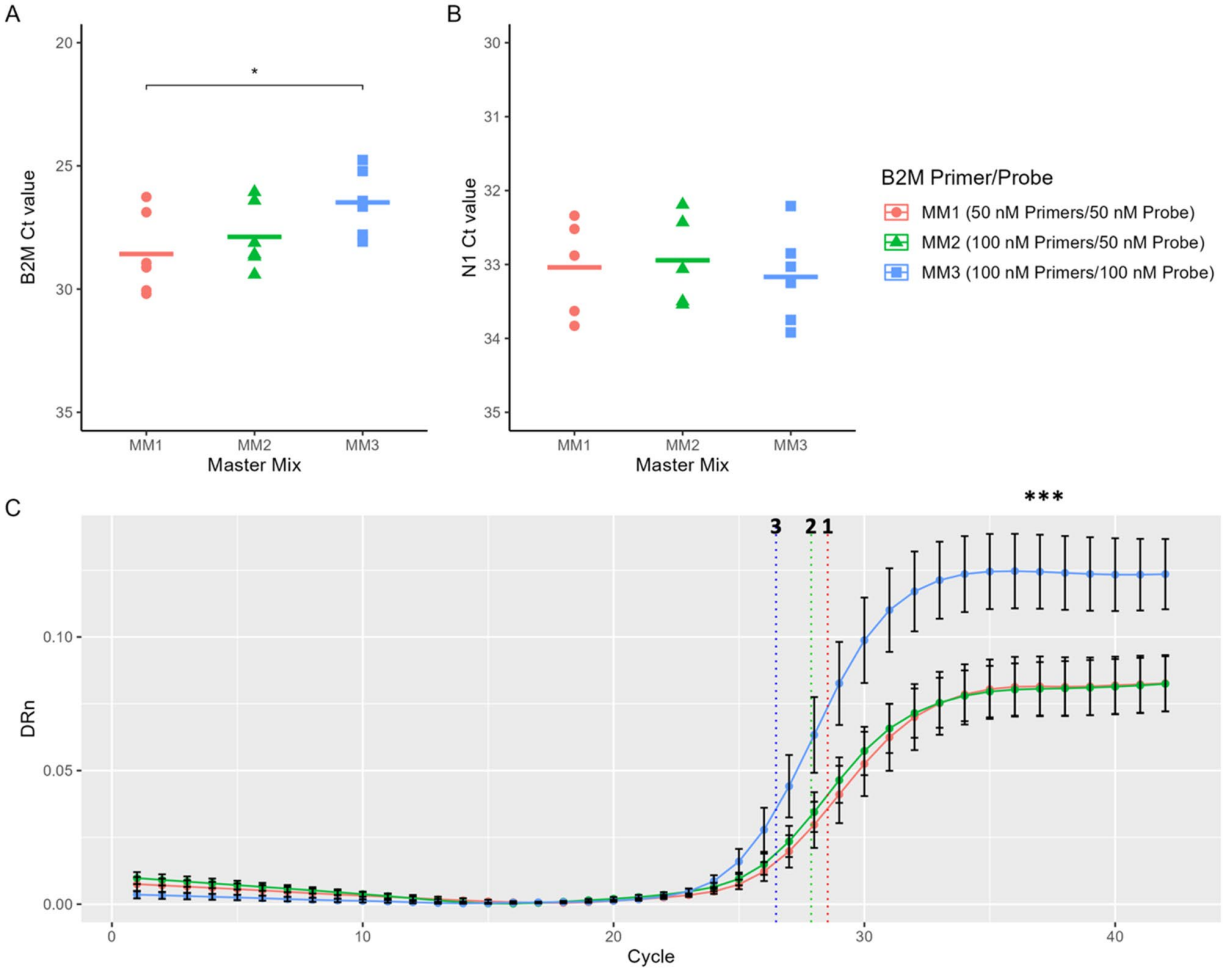
Optimizing the B2M primer/probe concentration. Previously confirmed N1 negative RNA samples (extracted by Acid-pH) were pooled together and amplified by q-RT PCR to detect the internal B2M control, using three master mix (MM) recipes. Each of these contained a different concentration of B2M primer/probe. (**A**) The Ct values of B2M and (**B**) N1 from PCRs using each of the three master mixes, each with varying concentrations of the B2M primer and probe. Average Ct values denoted by black bars. *Significant at p < 0.05, t test. (**C**) The B2M Delta Rn (DRn) values plotted against cycle numbers to generate the average B2M curves by RT-qPCR using each of the three master mix recipes. The average B2M Ct values from each of the three master mixes denoted as dotted vertical lines. ***Significant at p < 0.001, One way ANOVA. n = 3 experiments, 2 technical replicates per experiment. Error bars represent standard error of the mean.

Using Direct Lysis, B2M was positively detected 21 times out of the 58 samples tested (36.2% concordance). In contrast, the Concentrated Acid-pH protocol performed better, where B2M was positively detected 51 times out of the 58 samples tested (87.9% concordance). The Ct values of B2M were also compared across the method of extraction. It was observed that B2M Ct values were significantly higher in samples extracted by Direct Lysis than Concentrated Acid-pH (p < 0.001, Fig. 1B). For RNase P, the standard kit utilized was designed to be detected in the ABY channel. However, the instrument used was not equipped to detect the emission wavelength of the ABY dye, therefore both the HEX (Fig. 1C) and the ROX channels (Fig. 1D) were used to analyze fluorescence for these RT-qPCR assays. In the HEX channel, RNase P was positively detected 25 times out of the 58 samples tested by Direct Lysis (43.1% concordance). RNase P was detected in 53 of the 58 samples extracted with Concentrated Acid-pH (91.4% concordance) (Fig. 2B). The results in the ROX channel mimicked that seen in HEX. Concentrated Acid-pH once again performed well, with RNase P detected in 52 of the 58 samples (89.7% concordance). Detection of RNase P by Direct Lysis, however was only concordant in 17 of the 58 samples (29.3% concordance). Comparing Ct values across methods showed that in both the HEX and ROX channels, RNase P Ct values were significantly higher when the sample was extracted with Direct Lysis compared to Concentrated Acid-pH (p < 0.001, Fig. 1C,D). However, because RNase P was detected more times overall from both methods in the HEX channel it was concluded that using this channel was the most effective way to measure its fluorescence.

Lastly, the detection of RNase P and B2M was compared in Concentrated Acid-pH RNA extractions. While both were successful internal controls, the Ct values of B2M were significantly higher than those of RNase P (p < 0.001, Fig. 1E). Taken together these results suggested that the Concentrated Acid-pH Method was superior and RNase P the best internal control. This was therefore the protocol utilized during the initial months of this study.

### Optimization of Acid-pH and Direct Lysis methods

As work progressed, several iterations were made to the Acid-pH Method to further optimize (detailed in the Methods section). The primary change was to revert to the 1X lysis buffer however, now using only 100 μL rather than 300 μL as reported by Wozniak et al. Herein, the optimized Acid-pH protocol is referred to as the Acid-pH Method. The ability to use either B2M or RNase P as internal controls for the screening platform had been demonstrated (Fig. 1). However, the use of B2M rather than RNase P was more economical since RNase P detection relied on a commercially produced assay (Thermo Scientific) whereas Aumintec had designed the B2M primer/probe in-house. In practice, while the Ct values of B2M were adequate, the peak fluorescence was always much lower than in tests utilizing RNase P (data not shown). Therefore, the PCR reaction was further optimized to improve B2M detection. This would reduce the possibility of void tests due to a weak B2M signal. Three different PCR master mixes were tested, with master mix 1 (MM1) being composed of a 1:1 ratio of B2M primer/probe at a concentration of 50 nM each per reaction. This was the recipe used for the experiments in Fig. 1. MM2 contained a 2:1 ratio of B2M primer/probe with the concentration of primer increased to 100 nM per reaction. MM3 once again contained a 1:1 ratio of B2M primer/probe with a concentration of 100 nM per reaction of each. Across three experiments, the average B2M Ct value generated by MM1 was 28.54. The Ct values of B2M were lower in PCR reactions using MM2 and MM3, with values of 27.88 and 26.48, respectively. The average Ct value generated by MM3 was significantly lower than that of MM1 (p < 0.05, Fig. 2A). Next, different master mixes were evaluated to determine whether they affected the detection of the SARS-CoV-2 N1 sequence. For this, a standard positive template control (PTC) yielded average Ct values of N1 of 33.37, 32.44 and 33.17 for MM1, MM2 and MM3 respectively. These differences were not statistically significant (Fig. 2B). Lastly the RT-qPCR curves for B2M were compared across the different master mixes. It was found that peak fluorescence (graphed as DRn) was significantly higher when using MM3 rather than MM1 or MM2 (p < 0.001) (Fig. 2C). Therefore, B2M was deemed a more cost-effective and adequate performing internal control for saliva extract RT-qPCRs and was used exclusively moving forward.

**Figure 3:**
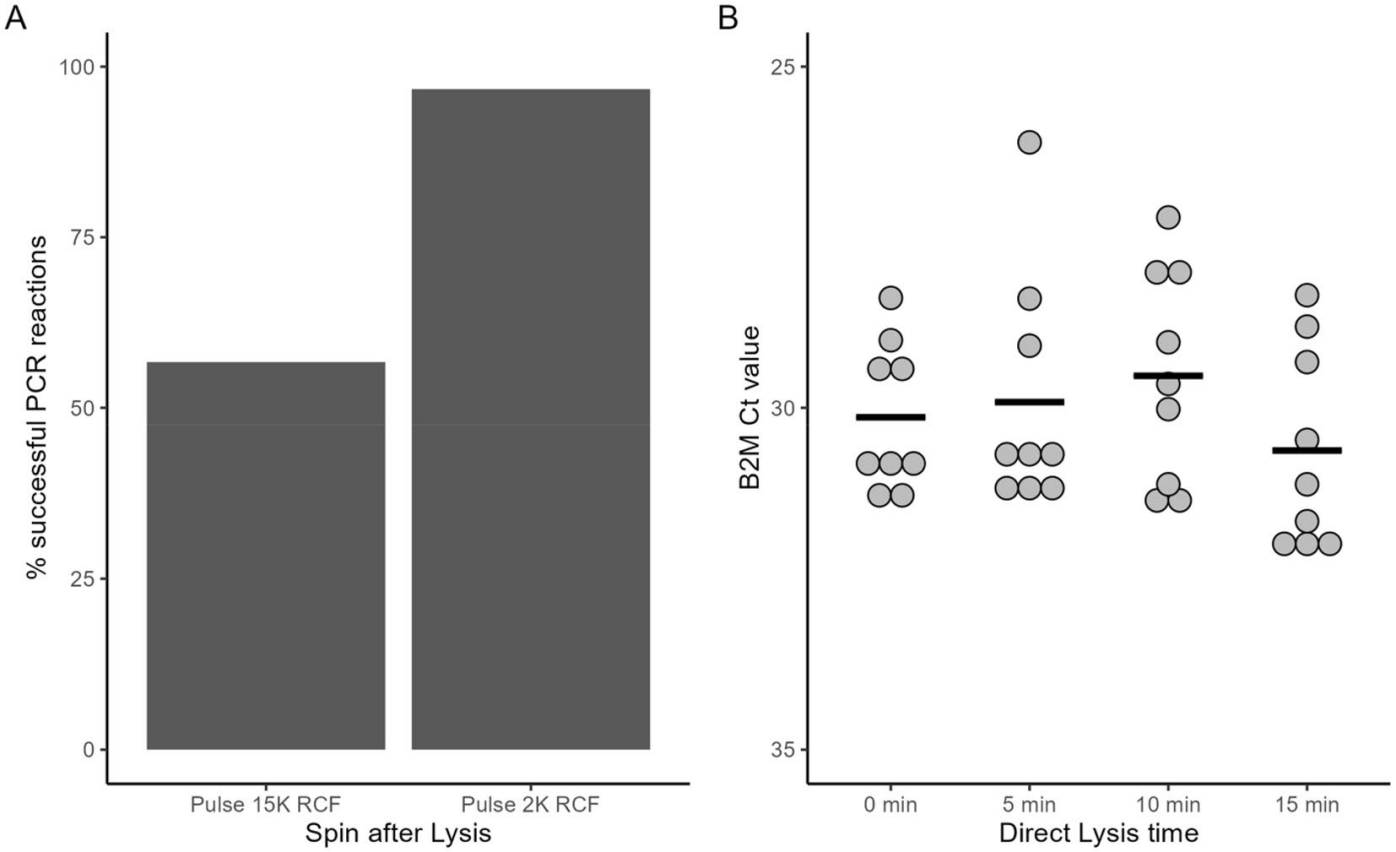
Direct Lysis method optimization. Direct Lysis was performed on saliva samples, and q-RT PCR was run in triplicate for each sample to detect B2M. A reaction was deemed successful if B2M was detected. (**A**) The difference in technique was either a fast (15,000 RCF) or slow pulse spin (~ 2000 RCF) after lysis. The percent concordance based on total PCR reactions. Fast spin, n = 40 direct lysis reactions, 120 PCR reactions measured. Gentle spin, n = 59 direct lysis reactions, 177 PCR reactions measured. (**B**) Previously confirmed (by Acid-pH Method) negative saliva samples were pooled together, and some of the pooled sample was subjected to direct lysis for 0, 5, 10, or 15 min before q-RT PCR to detect B2M internal control. The Ct value of B2M was not significantly affected by the duration of the direct lysis (One way ANOVA). n = 3 experiments, 3 technical replicates per experiment.

While Direct Lysis was technically capable of producing a product in which B2M (and RNase P) could be detected, it was by far an inferior method to the Acid-pH Method (Fig. 1). We wished to improve this extraction as Direct Lysis was useful to test participants who had not provided enough saliva for Acid-pH, or as an option for a quick test/retest if the result was particularly time-sensitive. The first change to the protocol was the addition of a quick pulse spin after the 5-min lysis step. However Direct Lysis concordance remained inconsistent in practice. To optimize this, two different spin conditions were tested. The first was a “fast” pulse spin. To do this, the samples were pulse spined until the centrifuge reached speeds of approximately 15,000 RCF. The second was a “gentle” pulse spin, where samples were pulsed up to speed no more than 2000 RCF. These samples were then tested for detection of B2M by RT-qPCR to gauge their concordance. The concordance of Direct Lysis was greatly improved with spinning before RT-qPCR. Previously, it was shown that the initial protocol yielded B2M detection of 36.2% (Fig. 1). B2M was detected in 56.7% of reactions when the samples were pulse spun until reaching a speed of 15,000 RCF. However, a tremendous improvement was observed with pulse spinning only up to 2000 RCF, with B2M now detected in 96.7% of reactions (Fig. 3A). We also tested whether the duration of the Direct Lysis affected the detection of B2M by RT-qPCR. Saliva samples were lysed for 15, 10, 5 and 0 min prior to running PCR and detection of the internal B2M control was used to gauge the effectiveness. The average Ct values of B2M were as follows: 30.63 (15 min), 29.53 (10 min), 29.92 (5 min) and 30.14 (0 min). These differences were not statistically significant, and we continued with our standard 5-min Direct Lysis time (Fig. 3B).

**Figure 4:**
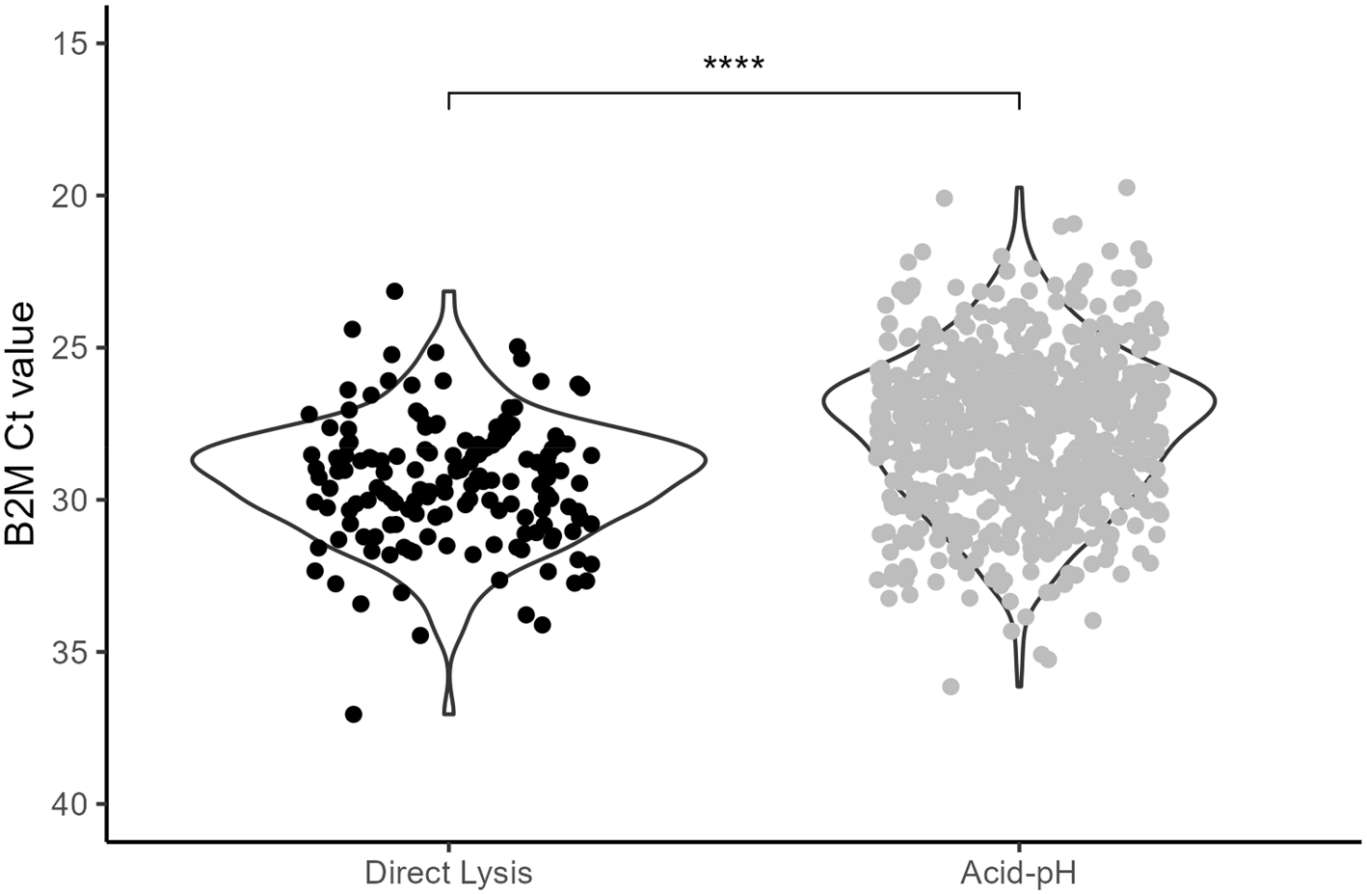
Optimized Acid-pH and Direct Lysis Methods. Saliva samples were extracted using the optimized versions of the Acid-pH and Direct Lysis Methods. The Ct values of B2M were compared. Acid-pH, n = 338 extractions, 676 PCR reactions. Direct Lysis, n = 57 extractions, 171 PCR reactions. ****Significant at p < 0.0001, t test.

**Figure 5:**
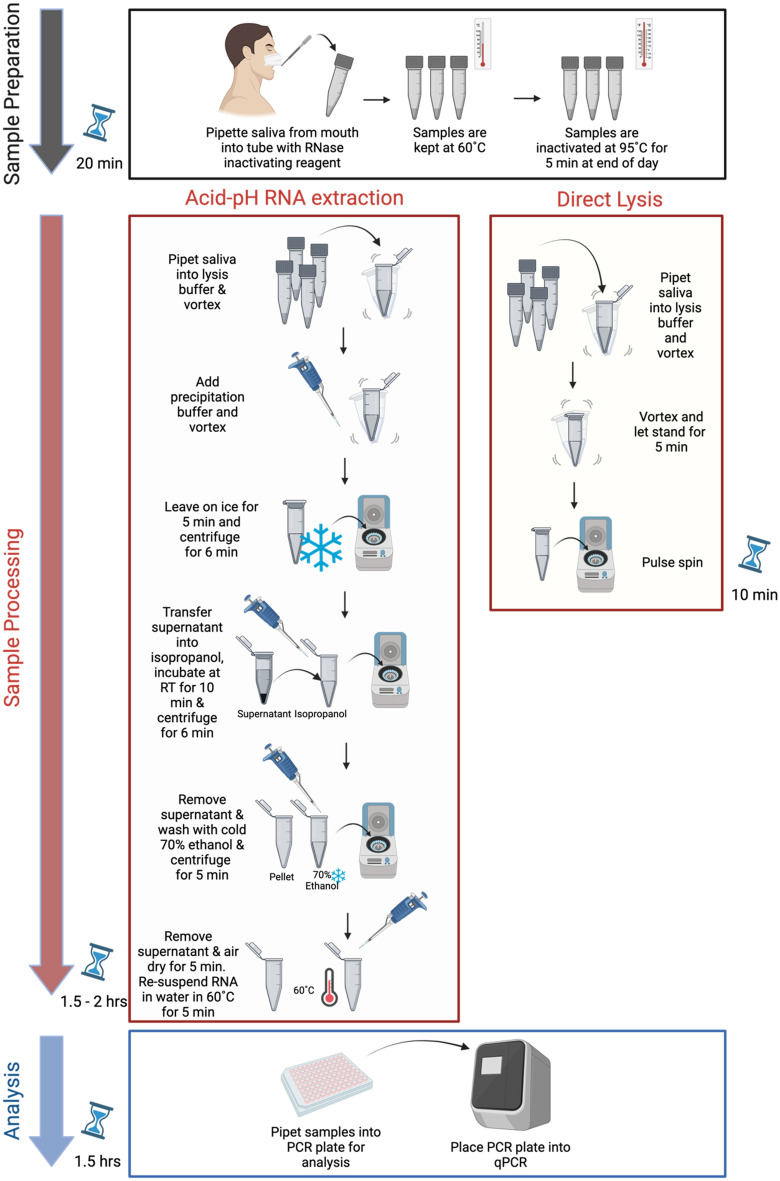
Summary of optimized Acid-pH RNA extraction and Direct Lysis. Preparation of saliva for processing takes 20 min. Acid-pH RNA Method can be completed in 1.5–2 h, depending on sample load. Direct Lysis Method can be completed within 10 min. PCR amplification of either extract will take 1.5 h. Created with BioRender.com.

With Acid-pH and Direct Lysis Methods optimized, we wished to compare these to each other once again. These results are seen in Fig. 4. With this sample group, Acid-pH was 96.9% concordant (676 of 697). These 676 reactions represent 338 saliva samples. Direct Lysis was performed on 57 saliva samples (171 PCR reactions). These were the successful “gentle” Direct Lysis reactions reported in Fig. 3A. The Ct values of B2M were compared to evaluate their performances. The mean B2M Ct value for Acid-pH was 27.68. In contrast, the mean B2M Ct values were significantly higher at 29.32 (p < 0.0001). While both methods were optimized to rarely fail (96.9% concordance for Acid-pH, 96.7% for Direct Lysis) the B2M Ct values were still higher with Direct Lysis. These represent the current methods used in practice at this time and Acid-pH remains our preferred extraction method, recognizing that Direct Lysis has distinct advantages especially when low volumes of sample are available. The protocols are compared and summarized in Fig. 5.

### Limit of detection (LOD) of SARS-COV-2 for Acid-pH and Direct Lysis methods

**Figure 6:**
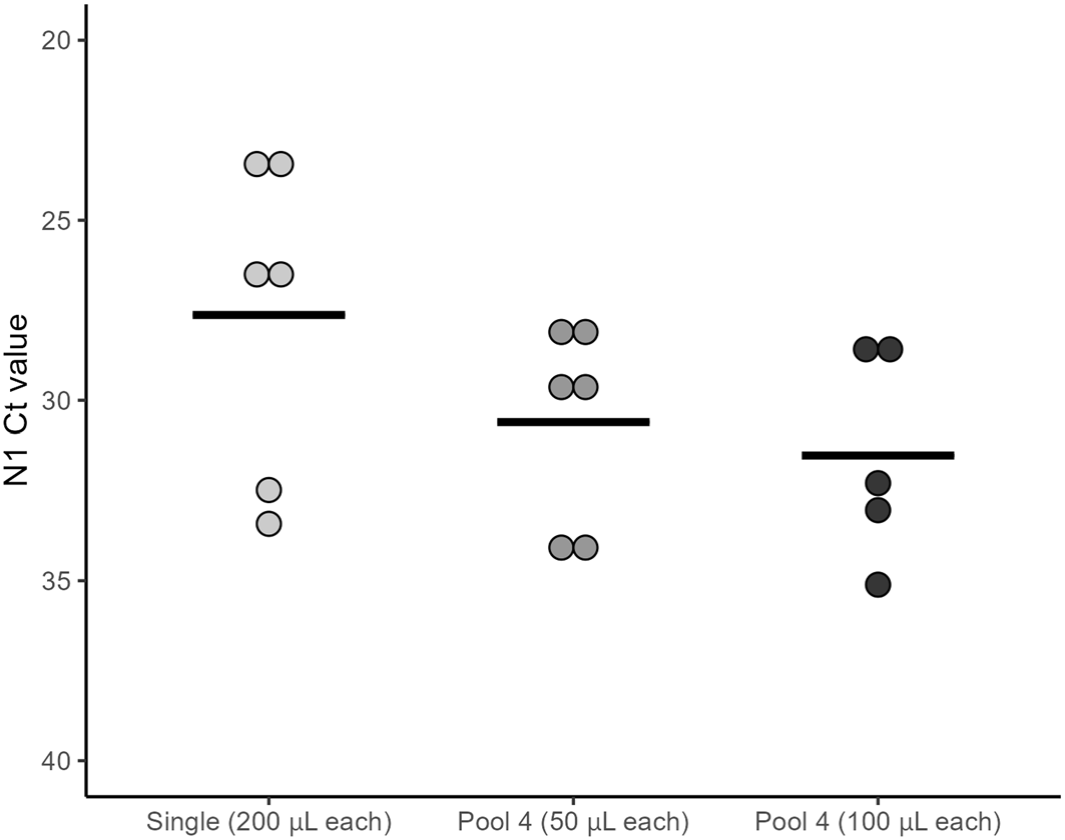
Detection of N1 from positive saliva in pools of 4. Previously identified positive saliva samples were pooled with three negative samples to create pools of 4. These were done with 50 μl each as well as 100 μl each. Acid-pH RNA extraction was performed on these pools, as well as the positive samples alone and the Ct values of N1 were compared. For the pool of 4 with 100 μl each, the volume of lysis buffer was increased to 200 μL to accommodate. n = 3 pools/single samples. 6 PCR reactions for single/Pool 4 50 μL, 5 PCR reactions for Pool 4 100 μL (ns, One way ANOVA).

Our data thus far shows that the Ct values by Direct Lysis are higher than those generated by the Acid-pH Method. A LOD assay for the detection of N1 was performed to quantify this. Negative saliva was spiked with commercial SARS-CoV-2 Viral-like particles (VLPs) to create samples with a known concentration of VLPs before extraction. The concentrations tested were 10, 5, 2.5, and 1 VLP copies/μL of saliva. These samples were processed with both the Acid-pH and Direct Lysis Methods, and 5 replicates were analyzed by RT-qPCR to detect N1. The LOD was the same for both Acid-pH and Direct Lysis at 10 copies/μL, with a concordance rate of 80% (4/5). The Ct values of N1 ranged from 33.37 to 34.86 for Acid-pH and 36.17 to 41.30 for Direct Lysis. The LODs at 5 copies/μL began to differ for the two extraction methods. Acid-pH was once again 80% successful on saliva with this VLP concentration. In contrast, Direct Lysis was only 20% successful (1/5) at 5 copies/μL. The Ct values for Acid-pH ranged from 34.05 and 35.96, while the only successful Direct Lysis reaction had a Ct value of 37.53. At both 2.5 and 1 copy/μL, Acid-pH and Direct Lysis Methods were both 20% successful. At 2.5 copies/μL, the Ct values were 38.14 and 36.13 respectively for Acid pH and Direct Lysis. The Ct values were 41.31 for Acid pH and 40.69 for Direct Lysis at 1 copy/μL (Fig. 6).

### Acid-pH analysis of pooled participant samples

**Figure 7:**
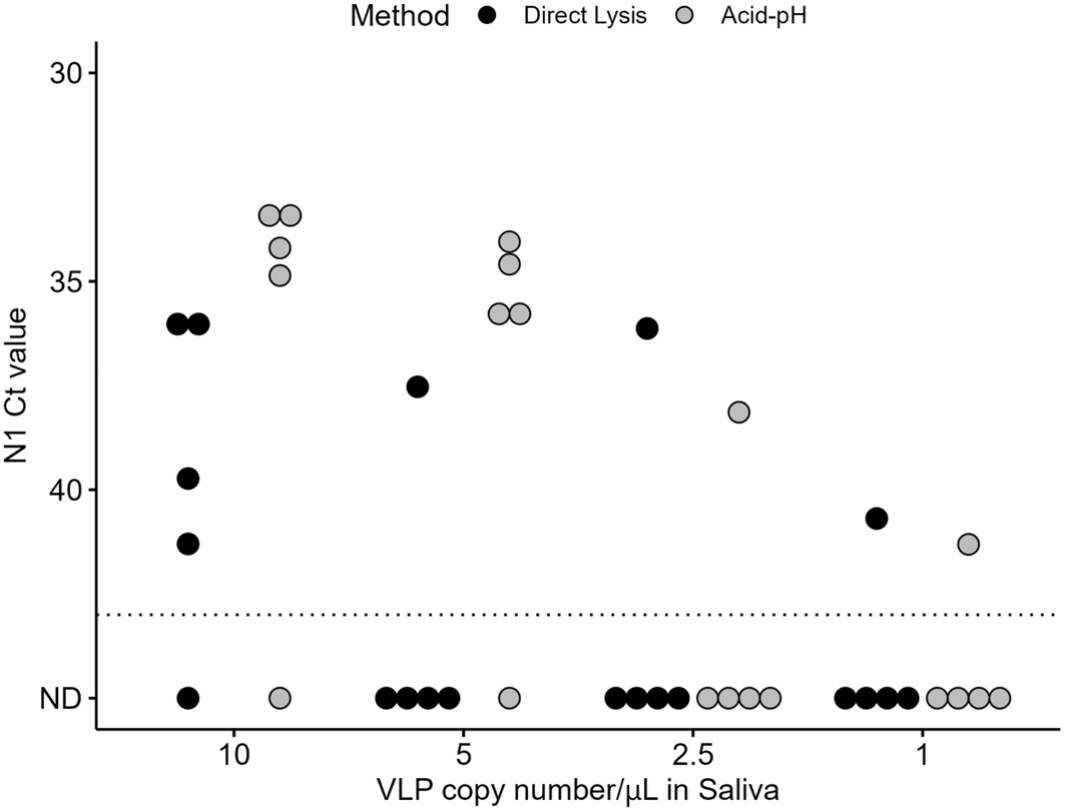
N1 limit of detection in acid-pH and Direct Lysis Methods. Saliva samples were spiked with commercial N1 viral-like particles (VLPs) to control for the copy number concentrations. 10, 5, 2.5 and 1 copy/μL saliva was created, processed by Acid-pH and Direct Lysis Methods, and run with RT-qPCR to detect N1. The dashed line represents the detection limit. n = 5 PCR replicates per extraction.

We next wished to determine the sensitivity of our methods using pooled saliva samples. This would then permit larger population-based screening, especially beneficial for asymptomatic detection of the virus. Three different saliva samples that had tested positive for COVID-19 as an individual sample were each pooled with saliva from three confirmed negative samples. This created 3 pooled samples containing 50 μL of saliva from 4 individuals, with one confirmed positive in each, for a total of 200 μL in each sample. Additionally, a pool of 4 was also created by using 100 μL of saliva from each participant for a total of 400 μL. For these latter samples, the amount of lysis buffer used was increased from 100 μL to 200 μL to accommodate the extra saliva. Otherwise, the Acid-pH protocol remained unchanged. Acid-pH Method was performed on the pools of 4, as well as the single positive samples, and the Ct values of N1 were compared. The average Ct values were 27.63, 30.61, and 31.52 for single positives, pool 4 (50 μL each) and pool 4 (100 μL each) respectively. While we found that the average Ct values increased in the pooled samples, these were not statistically significant (Fig. 7). These results demonstrate the ability to detect N1 in true positive cases in at least a pool of 4 samples.

### Detection of SARS-COV-2 over time in double vs. triple vaccinated individuals with both Acid-pH and Direct Lysis

**Figure 8:**
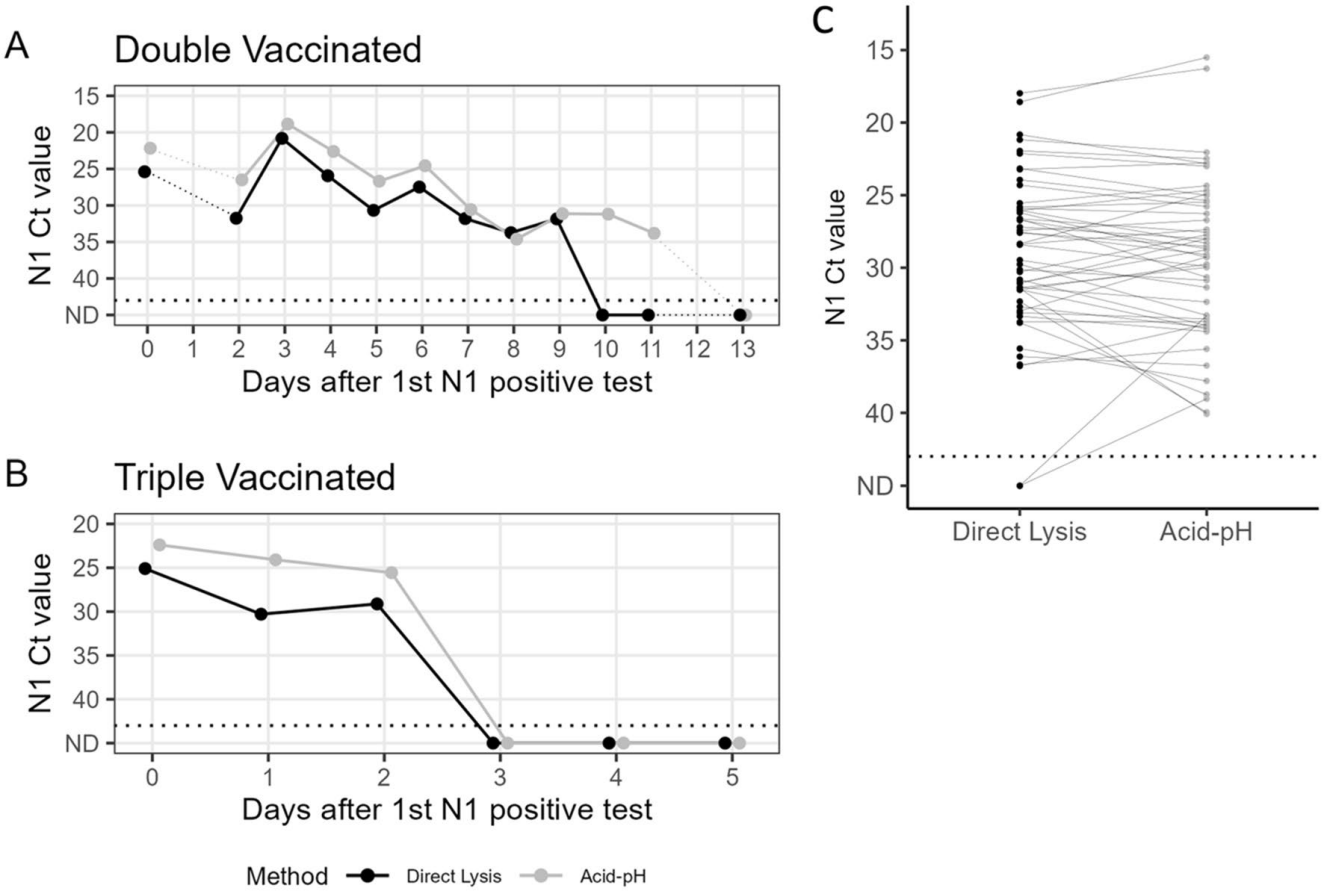
Case study comparison and SARS-CoV-2 positive saliva comparisons of acid-pH and Direct Lysis methods. Two cases where people tested positive and were able to collect their own saliva over multiple days, and the saliva was tested with both Acid-pH and Direct Lysis Methods. Each point represents the average Ct of N1 based on two PCR replicates. The individual in (**A**) was double vaccinated, with samples ranging from Day 0–13 days after N1 was initially detected in their saliva. No samples from Day 1 or Day 12 were received. The individual in (**B**) was triple vaccinated, where we had samples ranging from Day 0–5 days after N1 was initially detected in their saliva. (**C**) 51 SARS-CoV-2 positive saliva samples were processed with both methods to compare the Acid-pH and Direct Lysis methods. Each point represents the average Ct of N1 bsed on two PCR replicates (ns, t test).

Two participants who tested positive for COVID-19 volunteered to collect their own saliva remotely after being provided with collection tubes containing RNAsecure™. After testing negative by rapid antigen test as well as concluding their quarantine, the participants provided a series of saliva samples for testing. Collected samples were tested with both Acid-pH as well as Direct Lysis, as a final evaluation of these two methods together (Fig. 8). The first participant (Case 1) disclosed that they were double vaccinated. Their saliva samples spanned a course of 14 days (Day 0-Day 13 after initial test). This individual did not provide samples for Days 1 or 12. While the Ct values of N1 typically followed the same pattern across all days, they were consistently lower for the Acid-pH Method vs those from Direct Lysis, except for Day 9. Their peak viral load occurred on Day 3, based on the lowest N1 Ct values on this day. This was reflected in both the Acid-pH and Direct Lysis data sets. The most striking differences were observed between the samples from Days 10 and 11. On Day 10, N1 was detected in the RNA extract (Mean Ct 31.20), whereas it was not detected in Direct Lysis from that day’s sample. On Day 11, N1 continued to be detected in the RNA extract (Mean Ct 33.81) while still being absent from the Direct Lysis. On Day 13, N1 was not detected in both Acid- pH RNA and Direct Lysis extractions (Fig. 8A). The second of these two participants (Case 2) disclosed that they were triple vaccinated. Their saliva samples spanned a course of 6 days (Day 0-Day 5 after the initial test). This individual tested positive from Day 0 to Day 2. Their peak viral load was on Day 0, with the lowest Ct values detected reflected by both Acid-pH and Direct Lysis data sets. The Ct values of N1 were higher from Direct Lysis than Acid-pH RNA extraction in all three days. On Days 3 through Day 5, N1 was not detected in Acid-pH RNA or Direct Lysis extracts (Fig. 8B). The sequences of these samples and lineage delineation were later obtained by Nanopore sequencing and bioinformatic analysis using a modified version of the Galaxy server pipeline^36^ (Supplementary Methods S1). The first and second participants in Fig. 8 were determined to have been infected with Omicron BA.1 (Coverage = 95.6%, Phred quality score = 17.7) and Omicron BA.2 (coverage = 94.8%, Phred quality score = 22.5) variants respectively (Supplementary Fig. S1).

The two methods were also compared using 51 SARS-CoV-2 positive saliva samples. Direct lysis was unable to detect N1 in two samples where the Ct value for Acid-pH was 33.3 and 39.0, which was towards the end of the individuals’ course of infection. This is like what we see in the case study for individual A. Based on the t-test, there was no significant difference in the two methods for these SARS-CoV-2 positive saliva samples.

## Discussion

Collectively, these data chronicle the optimization of two extraction methods used on saliva that are suitable for the detection of the SARS-CoV-2 N1 sequence by q-RT PCR. Acid-pH and Direct Lysis methods are low-cost RNA extraction techniques that can be performed with common lab reagents and without the need for expensive RNA extraction kits (Supplementary Information S1). This is an advantage of our platform over others conducted at other universities that have used expensive kits and/or liquid-handling robots as part of their protocols^37–39^. This lowers the barrier to entry for setting up routine testing platforms that have a smaller budget.

The Acid-pH Method that was chosen for this saliva screening platform was adapted from the original method developed by 2/*9/2024* 2:46:00 PM. The lysis buffer used is at pH 5 which creates an environment where RNA degradation is reduced. The RNA phosphodiester backbone is more stable at acidic pH, however, is subject to alkaline hydrolysis at pH higher than 6^40^. Originally, the major modification introduced in this study was reducing the volume of lysis buffer and concentrating it 3X (Concentrated Acid-pH Methods). This would be advantageous for pooling many samples to facilitate population-based testing and processed in 2.0 mL tubes in microcentrifuges. When comparing the original lysis buffer concentration with the concentrated lysis buffer, the Ct values for the latter was significantly lower, p < 0.01. However, for the screening platform, the Concentrated Acid-pH Method successfully detected internal controls B2M and RNase P in 87.9% and 91.4% of samples, respectively. While promising, a failure of up to 12% is high for routine testing. Therefore, efforts to optimize the protocol continued. The optimized Acid-pH Method maintained the reduction of lysis buffer volume, however returned the concentration of the lysis buffer to 1X, as established previously^34^. The concordance rate of the Acid-pH tests was now 97%, 10% higher and more acceptable in a routine testing setting than the concentrated version. It is possible the concentrated method failed more due to the increased SDS in the lysis buffer. This detergent is a surfactant molecule that aggregates into micellar structures at higher concentrations. Above this critical micellar concentration (CMC), SDS may actually induce or stabilize the helical structures of proteins^41–43^. Therefore, 3X buffer may be approaching the CMC of SDS, possibly by inhibiting protein denaturation and lysis of the cells in some cases. However, our optimization demonstrated that the volume of the lysis buffer could still be reduced while maintaining the more effective 1X buffer for cell lysis.

The initial Direct Lysis Method yielded a low rate of concordance for detection of both B2M and RNase P, at 36.2 and 29.3% respectively. Saliva contains PCR inhibitors that are likely proteins that are present in salivary gland excretions^44^. It is possible that there was PCR interference in the crude Direct Lysis. Improvement of a DNA target may be achieved by boiling the saliva for at least 6 min^44^. Here, saliva samples were first heated for 10 min at 60 °C followed by 5 min at 95 °C to heat inactivate. Perhaps increasing this time from 6 to 8 min could improve this. It should be noted though that different gene targets may be affected by inhibitors differently and is something to consider in multiplex reactions^45^. The SPUD assay is an option to determine whether there are inhibitors present in the PCR reaction and should be considered when troubleshooting difficult PCR^46^. During further optimization, centrifugation of the samples post lysis was tested regarding the concordance of the PCR reaction. Direct Lysis concordance of 96.7% was achieved with a gentle pulse spin up to 2000 RCF before PCR. Amplification of samples after a faster pulse spin of 15,000 RCF was only 56.7% concordant. We speculate that some inhibitors present in the lysate are pelleted with a gentle spin speed, however, may aggregate RNA from the lysate if spun faster. It was determined however, that the duration of Direct Lysis did not affect the amplification of B2M significantly between 0 and 15 min. It has been shown that RT-qPCR can be successful in NPS swab samples as well as saliva by just heat inactivating and amplified directly^47,48^. Heat inactivation of saliva may be sufficient for effective lysis, and addition of lysis buffer only improves it. While not significant, the average B2M Ct values were trending lower from 0 to 10 min, possibly displaying this. Nonetheless, we report here a well optimized version of saliva Direct Lysis.

Acid-pH uses more saliva than Direct Lysis, theoretically concentrating more RNA in the final extract. A higher concentration of RNA in the sample will correlate with lower Ct values of the analyte sequence^46^. Additionally, Acid-pH is a true RNA extraction method. The steps of this protocol are designed to isolate pure RNA and exclude PCR inhibitors as discussed earlier. Direct Lysis in contrast is a one-step lysis method, where the crude lysate is used directly for PCR with no more than a pulse spin beforehand. It should be noted that in our practice, some of the Direct Lysis PCR results exhibited amplification artifacts in the FAM spectrum, in which we were using to detect N1. These were low Ct “curves” unlike what was expected with genuine N1 detection based on our positive template controls. Artifacts like these were also never present in matched Acid-pH extractions if performed. While it was distinct enough from the true signal in our workflow, this is still a disadvantage of Direct Lysis that potential users should be aware of.

Between the optimized Acid-pH RNA extraction and Direct Lysis, the former proved to be the better method based on the quality of the result. This was seen in the form of significantly lower Ct values of the B2M internal control in our large sample size. The average B2M Ct value for Acid-pH was 27.68 and for Direct Lysis it was 29.32. The limit of detection of Acid-pH was lower than Direct Lysis. Additionally, in positive cases, we observed lower N1 Ct values in Acid-pH extractions compared to the paired Direct Lysis. The positive samples also revealed that in samples with high N1 Ct values detected in Acid-pH RNA extracts, N1 may go undetected in the matched Direct Lysis. Therefore, there is the possibility of false negative results in these cases. Cases with the highest N1 Ct values may represent those at the end of their infection. These would be individuals who may test positive by PCR, however, are negative for the viral antigen at this point and not infectious^11^. They may also represent individuals right at the beginning of their infection. Reporting these false negatives could be problematic, however, with stronger extraction methods like Acid-pH and, most importantly more frequent testing, these occurrences can be minimized. In our case study, we present data tracking the Ct values of N1 over multiple days after an initial positive test. The individual who was double vaccinated exhibited N1 detection for 12 days, whereas the boosted individual was positive for only 3 days. Sequencing of these RNA samples revealed Case 1 to be Omicron BA.1. and Case 2 to be BA.2 One BA.1 study in fully vaccinated and boosted individuals showed that there was a significant decrease in infectious viral load but not RNA viral load (as measured by RT-qPCR). It should be noted however that measurements were only made up to 5 days post onset of symptoms. And while the RNA viral loads were not significantly lower, there were no measurements detected on Day 5 in boosted individuals^2^. This is consistent with our case study, however, time course sampling in more positive cases would be needed before any conclusions could be fully formed.

The advantages of the Acid-pH extraction led to the conclusion that it should be the preferred extraction method for testing. However, this method is not without its drawbacks. Acid-pH RNA extraction requires 200 μL of saliva, whereas Direct Lysis can be done with 56 μL. This is useful in cases where the participant did not provide sufficient saliva for Acid-pH but can still be tested by Direct Lysis. Perhaps the largest advantage of Direct Lysis is time, which only takes 5 min. Acid-pH RNA extraction requires between 1.5 and 2 h to complete depending on the volume of samples being processed. Therefore, Direct Lysis could be especially useful for a quick re-test using the remainder of a participant’s sample, or for more time sensitive turnaround. “Saliva Direct”, a method like Direct Lysis was used in partnership with the National Basketball Association (NBA) where quick results were required for minimal interruptions. The authors however stated that there was a minimal, yet significant increase in Ct values compared to RNA extraction, as we have noted with our Direct Lysis^4^. While Acid-pH extraction produces better results, both methods in most cases are reliable. The advantages and disadvantages of Acid-pH and Direct Lysis may hold different weight based on the needs of the testing platform, and one may be the preferred method based on these unique criteria.

While the NPS swab has been the gold standard for COVID-19 testing through the pandemic, it has been replaced in some cases by the Midturbinate swab (MTS). The swab is not inserted as far into the nasal cavity with MTS and hence is slightly less invasive, creating less discomfort for the person being tested, and requires less training. MTS however compromises on the sensitivity, being notably less sensitive than NPS^49^. Swabbing in general also comes with the risk of infection for the medical professional by means of droplets and aerosols^50^. Therefore, the use of saliva instead of any form of swabbing may be more advantageous. This setup requires no trained medical personnel. The participant can deposit their saliva sample independently, with no contact with anyone during this process and the saliva is heat inactivated before any exposure to lab personnel. Pooling of saliva is possible as well, and we have shown that N1 is still detectable in pools from up to 4 individuals. Interestingly, saliva may also be a better method for early detection of COVID-19. Testing of saliva has been found to be most sensitive within 1–5 days of symptom onset^51^. Additionally, viral RNA was detected 12 times more in saliva compared to MTS. This was correlated with a 3.2% higher viral load^49^.

The use of saliva is not without its disadvantages. One concern is the quality of the participants' saliva which can be altered based on behavioral factors such as the timing of last consumption of food or drink prior to sampling. One meta-analysis determined that studies that instructed participants to not eat, drink or brush their teeth had an overall higher percent positivity detected than those that did not^52^. A study of potential confounding factors in saliva testing also identified mouthwash having affected the detection of 60% of the housekeeping genes tested^35^. While we did provide these instructions to the study participants, it is possible that they may not have always adhered strictly to them. Another factor to consider is that RNA extracted from saliva will be predominantly from the oral microbiome^53^. When targeting a more unique sequence like those from SARS-CoV-2, this is less likely to be a problem but may be a concern if the target sequences are certain microbial genes or low abundance human sequences.

Further innovations are being developed that can concentrate and detect viral antigens from saliva, which may be ideal for pooling many samples^54^. Miniaturized label-free electrochemical sensors and paper-based antigen sensing represent new alternatives for point-of-care or at-home testing^55,56^. These examples broaden the range of saliva testing beyond PCR amplification Saliva therefore represents an underutilized sample source that should be considered moving forward with COVID-19 and any other emerging viral pathogens of concern.

## Conclusion

Saliva is a non-invasive mode of sample collection that we have used to routinely screen a population safely for COVID-19. Here we have reported on the optimization and use of two different saliva extraction techniques for RT-qPCR testing, Acid-pH and Direct Lysis. These methods could be expanded to include screening for other pathogens of interest and represent low-cost options for these platforms.

### Supplementary Information

Supplementary Information.

## Data Availability

The data sets generated during and/or analysed are available from the corresponding author on reasonable request.

## References

[1] Riemersma, K. K. *et al. Shedding of infectious SARS-CoV-2 despite vaccination*. 10.1101/2021.07.31.21261387(2021).

[2] Puhach, O. *et al.* Infectious viral load in unvaccinated and vaccinated individuals infected with ancestral, Delta or Omicron SARS-CoV-2. *Nat. Med.* 1–1. 10.1038/s41591-022-01816-0 (2022). 10.1038/s41591-022-01816-0 35395151

[3] Bradley EH, An M-W, Fox E (2020). Reopening colleges during the coronavirus disease 2019 (COVID-19) pandemic—one size does not fit all. JAMA Netw. Open.

[4] Vogels CBF (2020). SalivaDirect: A simplified and flexible platform to enhance SARS-CoV-2 testing capacity. Medicine.

[5] Brook CE, Northrup GR, Ehrenberg AJ, Doudna JA, Boots M (2021). Optimizing COVID-19 control with asymptomatic surveillance testing in a university environment. Epidemics.

[6] Corchis-Scott R (2021). Averting an outbreak of SARS-CoV-2 in a university residence hall through wastewater surveillance. Microbiol. Spect..

[7] Scott LC (2021). Targeted wastewater surveillance of SARS-CoV-2 on a university campus for COVID-19 outbreak detection and mitigation. Environ. Res..

[8] Levy JI, Andersen KG, Knight R, Karthikeyan S (2023). Wastewater surveillance for public health. Science.

[9] Barrera-Avalos C (2021). The rapid antigen detection test for SARS-CoV-2 underestimates the identification of COVID-19 positive cases and compromises the diagnosis of the SARS-CoV-2 (K417N/T, E484K, and N501Y) variants. Front. Public Health.

[10] Osterman A (2022). Impaired detection of omicron by SARS-CoV-2 rapid antigen tests. Med. Microbiol. Immunol..

[11] Drain PK (2022). Rapid diagnostic testing for SARS-CoV-2. N. Engl. J. Med..

[12] Senok A (2020). Saliva as an alternative specimen for molecular COVID-19 testing in community settings and population-based screening. IDR.

[13] Tan SH, Allicock O, Armstrong-Hough M, Wyllie AL (2021). Saliva as a gold-standard sample for SARS-CoV-2 detection. Lancet Respir. Med..

[14] Balamane M (2010). Detection of HIV-1 in saliva: Implications for case-identification, clinical monitoring and surveillance for drug resistance. Open Virol. J..

[15] Chen KM (2013). Human papilloma virus prevalence in a multiethnic screening population. Otolaryngol. Head Neck Surg..

[16] Kwok H, Chan KW, Chan KH, Chiang AKS (2015). Distribution, persistence and interchange of Epstein–Barr Virus strains among PBMC, plasma and saliva of primary infection subjects. PLOS ONE.

[17] Pinninti SG (2015). Comparison of saliva PCR assay versus rapid culture for detection of congenital cytomegalovirus infection. Pediatr. Infect. Dis. J..

[18] Bonaldo MC (2016). Isolation of infective Zika virus from urine and saliva of patients in Brazil. PLOS Neglect. Trop. Dis..

[19] Podzimek S, Vondrackova L, Duskova J, Janatova T, Broukal Z (2016). Salivary markers for periodontal and general diseases. Dis. Mark..

[20] Kaczor-Urbanowicz KE (2017). Saliva diagnostics—Current views and directions. Exp. Biol. Med. (Maywood).

[21] Matuck BF (2021). Salivary glands are a target for SARS-CoV-2: a source for saliva contamination. J. Pathol..

[22] Azzi L (2020). Saliva is a reliable tool to detect SARS-CoV-2. J. Infect..

[23] To KK-W (2020). Temporal profiles of viral load in posterior oropharyngeal saliva samples and serum antibody responses during infection by SARS-CoV-2: An observational cohort study. Lancet Infect. Dis..

[24] De Santi C (2021). Concordance between PCR-based extraction-free saliva and nasopharyngeal swabs for SARS-CoV-2 testing. HRB Open Res..

[25] Johnson AJ (2021). Saliva testing is accurate for early-stage and presymptomatic COVID-19. Microbiol. Spectr..

[26] Marais G (2022). Improved oral detection is a characteristic of Omicron infection and has implications for clinical sampling and tissue tropism. J. Clin. Virol..

[27] Salmona M (2022). Detection of SARS-CoV-2 in saliva and nasopharyngeal swabs according to viral variants. Microbiol. Spect..

[28] Barat B (2021). Pooled saliva specimens for SARS-CoV-2 testing. J. Clin. Microbiol..

[29] Vander Schaaf, N. A. *et al.* Routine, cost-effective SARS-CoV-2 surveillance testing using pooled saliva limits viral spread on a residential college campus. *Microbiol. Spect.* **9**, e01089–21 (2021). 10.1128/Spectrum.01089-21 PMC8515933 34643445

[30] Fábryová H, Celec P (2014). On the origin and diagnostic use of salivary RNA. Oral. Dis..

[31] Abdalhamid B (2020). Assessment of specimen pooling to conserve SARS CoV-2 testing resources. Am. J. Clin. Pathol..

[32] Harris PA (2009). Research electronic data capture (REDCap)—A metadata-driven methodology and workflow process for providing translational research informatics support. J. Biomed. Inf..

[33] Harris PA (2019). The REDCap consortium: Building an international community of software platform partners. J. Biomed. Inf..

[34] Wozniak A (2020). A simple RNA preparation method for SARS-CoV-2 detection by RT-qPCR. Sci. Rep..

[35] Ostheim P (2022). Examining potential confounding factors in gene expression analysis of human saliva and identifying potential housekeeping genes. Sci. Rep..

[36] Afgan E (2018). The Galaxy platform for accessible, reproducible and collaborative biomedical analyses: 2018 update. Nucleic Acids Res..

[37] Avendano C (2022). SARS-CoV-2 variant tracking and mitigation during in-person learning at a Midwestern University in the 2020–2021 school year. JAMA Netw. Open.

[38] Ehrenberg AJ (2021). Launching a saliva-based SARS-CoV-2 surveillance testing program on a university campus. PLOS ONE.

[39] Hamilton JR (2021). Robotic RNA extraction for SARS-CoV-2 surveillance using saliva samples. PLOS ONE.

[40] Jarvinen P, Oivanen M, Lonnberg H (1991). Interconversion and phosphoester hydrolysis of 2’,5’- and 3’,5’-dinucleoside monophosphates: kinetics and mechanisms. J. Org. Chem..

[41] Khan JM (2014). Protonation favors aggregation of lysozyme with SDS. Soft Matter..

[42] Jafari M, Mehrnejad F (2016). Molecular insight into human lysozyme and its ability to form amyloid fibrils in high concentrations of sodium dodecyl sulfate: A view from molecular dynamics simulations. PLOS ONE.

[43] Jafari M, Mehrnejad F, Rahimi F, Asghari SM (2018). The molecular basis of the sodium dodecyl sulfate effect on human ubiquitin structure: A molecular dynamics simulation study. Sci. Rep..

[44] Ochert AS, Boulter AW, Birnbaum W, Johnson NW, Teo CG (1994). Inhibitory effect of salivary fluids on PCR: Potency and removal. Genome Res..

[45] Ståhlberg A, Zoric N, Aman P, Kubista M (2005). Quantitative real-time PCR for cancer detection: The lymphoma case. Expert Rev. Mol. Diagn..

[46] Nolan T, Hands RE, Bustin SA (2006). Quantification of mRNA using real-time RT-PCR. Nat. Protoc..

[47] Ranoa, D. R. E. *et al.* Saliva-based molecular testing for SARS-CoV-2 that bypasses RNA extraction. *bioRxiv* 2020.06.18.159434. 10.1101/2020.06.18.159434 (2020).

[48] Smyrlaki I (2020). Massive and rapid COVID-19 testing is feasible by extraction-free SARS-CoV-2 RT-PCR. Nat. Commun..

[49] Lai J (2022). Comparison of saliva and midturbinate swabs for detection of SARS-CoV-2. Microbiol. Spect..

[50] Qian Y (2020). Safety management of nasopharyngeal specimen collection from suspected cases of coronavirus disease 2019. Int. J. Nurs. Sci..

[51] Uddin MKM (2021). Diagnostic performance of self-collected saliva versus nasopharyngeal swab for the molecular detection of SARS-CoV-2 in the clinical setting. Microbiol. Spect..

[52] Lee RA, Herigon JC, Benedetti A, Pollock NR, Denkinger CM (2021). Performance of saliva, oropharyngeal swabs, and nasal swabs for SARS-CoV-2 molecular detection: A systematic review and meta-analysis. J. Clin. Microbiol..

[53] Ostheim P (2020). Overcoming challenges in human saliva gene expression measurements. Sci. Rep..

[54] Chen, Y., Liu, F. & Lee, L. P. Quantitative and ultrasensitive in situ immunoassay technology for SARS-CoV-2 detection in saliva. *Sci. Adv.* **8**, eabn3481 (2022). 10.1126/sciadv.abn3481 PMC9132547 35613342

[55] Farsaeivahid N, Grenier C, Nazarian S, Wang ML (2023). A rapid label-Free disposable electrochemical salivary point-of-care sensor for SARS-CoV-2 detection and quantification. Sensors.

[56] Jaewjaroenwattana J, Phoolcharoen W, Pasomsub E, Teengam P, Chailapakul O (2023). Electrochemical paper-based antigen sensing platform using plant-derived monoclonal antibody for detecting SARS-CoV-2. Talanta.

