# Supplementary material for: Evaluating and optimizing Acid-pH and Direct Lysis RNA extraction for SARS-CoV-2 RNA detection in whole saliva
Source: Sci Rep. 2024 Mar 25;14:7017. doi: 10.1038/s41598-024-54183-w (PMC10963802; doi:10.1038/s41598-024-54183-w)
Supplement: Supplementary file 1 — Supplementary Information. [file 41598_2024_54183_MOESM1_ESM.docx]

# Supplementary Methods

## Sequencing and Bioinformatics

Library preparation was performed employing the NEBNext ARTIC SARS-CoV-2 Companion Kit (Oxford Nanopore Technologies, Oxford, UK) following the nCoV-2019 sequencing protocol v3 ^1^ with modifications. For reverse-transcription,200-500 ng of RNA in 16 μL were mixed with 4 μL of LunaScript RT SuperMix (New England Biolabs, Ipswich, MA, USA) and incubated at 25 °C for 5 min followed by an incubation at 55 °C for 20 min. For cDNA amplification, the SARS-CoV-2 specific version 3 (V3) or VarSkip Short v2 (VSS2) primer sets designed by ARTIC Network were used. Multiplex-polymerase chain reaction was performed for each of both SARS-CoV-2 primer pools mixing 6.25 μL of Q5^®^ Hot Start High-Fidelity 2× Master Mix and 4.5 μL of the cDNA template with either 1.75 μL of primer pool #1 or primer pool #2. PCR reactions were carried out in duplicate. The mix was incubated at 98 °C for 30 sec followed by 35 cycles at 95 °C for 15 sec and 63 °C for 5 min before holding at 4 °C. PCR products were pooled and cleaned by adding 0.8 volume of NEBNext Sample Purification Beads (New England Biolabs). Sample was eluted in 15 μL nuclease-free water and quantified using a spectrophotometer (DS-11; DeNovix Inc., Wilmington, DE, USA). The end-prep reaction was performed employing 100 ng of PCR product mixed with 1.75 μL Ultra II End Prep Buffer (New England Biolabs), 0.75 μL Ultra II End Prep Enzyme (New England Biolabs) and water to a final volume of 20 μL. The reaction was incubated at 20 °C for 10 min followed by 65 °C for 10 min and holding at 4 °C. Barcoding of samples was carried out combining 3 μL of end-prepped sample, 2.5 μL Native Barcode (native barcoding EXP-NBD104, Oxford Nanopore Technologies), 10 μL Blunt/TA Ligase Master Mix (New England Biolabs) and 4.5 μL nuclease-free water. The ligation reaction was incubated at 22 °C for 20 min followed by 65 °C for 10 min and a hold on ice for at least 1 min. Cleaning of the ligated sample was performed by adding 0.4 volume NEBNext^®^ Sample Purification Beads (New England Biolabs) and eluting with 15 μL of nuclease-free water. Oxford Nanopore sequencing adaptor ligation was performed employing 200 ng of barcoded DNA in 50 μL, mixed with 5 μL Adapter Mix II, 10 μL 5× NEBNext Quick Ligation Reaction Buffer (New England Biolabs) and 5 μL Quick T4 DNA Ligase (New England Biolabs). The incubation was carried out at 25 °C for 20 min. Sample was cleaned by adding 1 volume of NEBNext Sample Purification Beads (New England Biolabs), eluted in 12 μL of elution buffer and quantified. Twenty ng of the library was loaded onto a minION Spot-ON Flow Cell (Oxford Nanopore Technologies).

A modified version of the bioinformatic pipeline available from Galaxy server ^2^ “*COVID-19: variation analysis of ARTIC ONT data”* was employed to analyze sequences. This pipeline facilitated rapid provision of pre-process sequences from FASTQ files. Mapping reads was carried out with minimap2 tool. Samtools, iVar trim and QualiMap BamQC were employed to convert between alignment formats, trim primer sequences and evaluate the quality of mapped reads, respectively. Medaka consensus and medaka variant tools were used to create a consensus sequence from Nanopore sequencing data and decode variant calls. Genetic variant annotation and effect prediction were carried out through bcftools and SnpEff toolbox. Consensus sequence generated by applying VCF variants (bcftools consensus) were further uploaded into the Pangolin web application to determine the dominant variant in the sample ^3^.

### Sequencing and Bioinformatics Results:

A


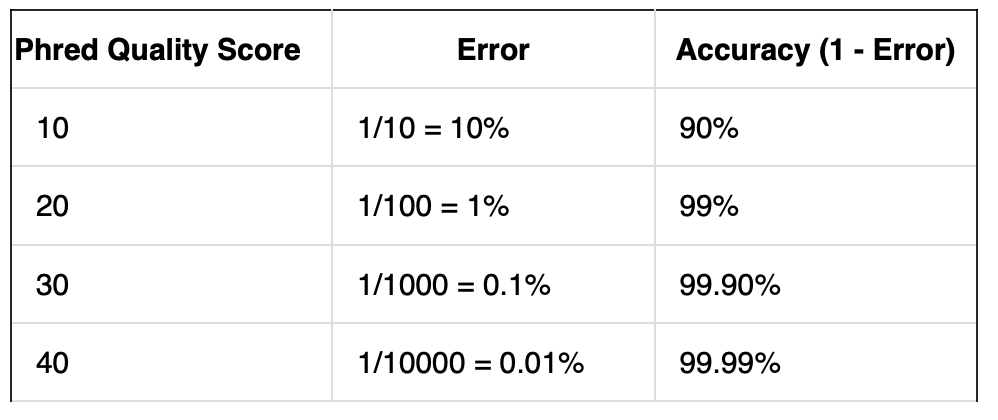


B

| **Sample** | **Primer pools** | **Average length of reads** | **Phred quality score** | **% GC** | **Error rate** | **% Coverage (>30X)** | **Median coverage** | **Mean coverage** | **Reads mapped (Million)** | **Clade** | **lineage** | **conflict** | **scorpio_call** | **scorpio_support** | **qc_notes** | **note** |
| --- | --- | --- | --- | --- | --- | --- | --- | --- | --- | --- | --- | --- | --- | --- | --- | --- |
| F25 | VSS2 | 621 | 17.7 | 41 | 11.48% | 95.6 | 5088.0X | 729.4X | 0.6 | 21K | BA.1.1 | 0 | Omicron (BA.1-like) | 0.76 | Ambiguous_content:0.04 | Usher placements: BA.1.1(1/1) |
| Case 2, April 14 (B1) | V3 | 488 | 22.5 | 40 | 5.63% | 94.8 | 3011.0X | 509.3X | 0.8 | 21L | BA.2 | 0 | Omicron (BA.2-like) | 0.92 | Ambiguous_content:0.05 | Usher placements: BA.2(1/1) |

**Supplementary Figure 1: Sequencing results and parameters for case study saliva samples.**

Data provided for the two individual case study participants. (A) Case 1 (double vaccinated) was referred to as F25 in the sequencing pipeline. Case 2 (triple vaccinated) was named appropriately. Phred quality score: Phred-scaled quality scores are used to represent how confident we are in the assignment of each base call by the sequencer. %GC: Mean GC content   % Coverage (>30X): Percentage of genome with at least 30X coverage (B) Error and accuracy percentages according to Phred quality score.

**Supplementary References**

1. Quick, J. nCoV-2019 sequencing protocol v3 (LoCost). (2020).

2. Afgan, E. *et al.* The Galaxy platform for accessible, reproducible and collaborative biomedical analyses: 2018 update. *Nucleic Acids Res* **46**, W537–W544 (2018).

3. O’Toole, Á. *et al.* Assignment of epidemiological lineages in an emerging pandemic using the pangolin tool. *Virus Evolution* **7**, veab064 (2021).

## Cost Analysis of saliva sample collection and q-RT PCR assay

The cost of the assay is presented for each part of the process, from saliva sample collection, sample processing and q-RT PCR amplification. All values indicated are in Canadian dollars.

**Saliva Sample Collection**

| **Item** | **Vendor** | **Cat No.** | **Quantity/**  **unit** | **Cost** | **Cost Per 100 samples** |
| --- | --- | --- | --- | --- | --- |
| General Purpose Transfer Pipets (Sterile, individually wrapped) | VWR | 414004-030 | 500 | $ 108.97 | $ 21.79 |
| MTC Bio SureSeal™ 2.0mL w/ loop caps, sterile, w/ O-ring, printed graduations, self-standing, caps assembled | Diamed | MTCC3220-SG | 500 | $ 269.00 | $ 53.80 |
| RNAsecure RNase Inactivation reagent | Thermo | AM7006 | 10 mL | $ 337.31 | $ 84.33 |
| **Total per 100 samples:** | | | | | **$ 159.92** |

**Sample Processing**

- **Acid-pH RNA Extraction**

The reagents used to make solutions for RNA extraction are presented as the costs to buy the reagents to make the buffers needed for the protocol.

| **Item** | **Vendor** | **Cat No.** | **Quantity/**  **unit** | **Cost** |
| --- | --- | --- | --- | --- |
| Sodium dodecyl sulfate (SDS) | BioBasic | SB0485 | 100 g | $ 27.80 |
| Sodium citrate dihydrate | BioBasic | CB0035 | 500 g | $ 18.34 |
| Citric acid anhydrous | Sigma | CX1723-1 | 500 g | $ 109.61 |
| EDTA | BioBasic | EB0185 | 500 g | $ 31.96 |
| Sodium Chloride | ACPChemicals | S2830 | 500 g | $ 48.69 |
| Ethanol | N/A | N/A | 2.5 L | $ 16.34 |
| Isopropanol | N/A | N/A | 2.5 L | $ 14.66 |
| 1.5 mL microcentrifuge tubes | VWR | 10011-700 | 500 | $ 19.59 |
| **Total Cost:** | | | | **$ 286.99** |

## Direct Lysis Method

The reagents used to make solutions for the RNA extraction are presented as the costs to buy the reagents to make the direct lysis buffer.

| **Item** | **Vendor** | **Cat No.** | **Quantity/**  **unit** | **Cost** |
| --- | --- | --- | --- | --- |
| IGEPAL® CA-630 | Sigma Aldrich | I8896-50ML | 50 mL | $ 85.11 |
| Sodium Citrate | BioBasic | CB0035 | 500 g | $ 18.34 |
| Tris-HCl | BioBasic | TB0195 | 500 g | $ 36.13 |
| **Total Cost:** | | | | **$ 139.58** |

**PCR Amplification**

| **Item** | **Vendor** | **Cat No.** | **Quantity/**  **unit** | **Cost** | **Cost Per 100 samples** |
| --- | --- | --- | --- | --- | --- |
| 0.2mL PCR 8-Strip Tubes with Individually Attached Optical qPCR Caps, Rigid | Ultident | 87-C200-8RGD-IND | 120 tubes | $ 79.95 | $ 133.25 |
| Takyon™ Dry One-Step RT Probe MasterMix No Rox | Eurogentec | UFD-NPRT-C0101 | 100 reactions | $ 241.80 | $ 483.60 |
| N1 Forward Primer | IDT | 10006830 | 100 µM in 1000 µL | $ 356.76 | $ 3.21 |
| N1 Reverse Primer | IDT | 10006831 | 101 µM in 1000 µL | $ 356.76 | $ 3.21 |
| N1 Probe | IDT | 10006832 | 50 µmol in 500 µL | $ 672.17 | $ 8.07 |
| Magnesium Chloride (MgCl2) Solution | New England Biobasics | B9021S | 25 mM in 6 mL | $ 35.37 | $ 0.11 |

- **Internal Control – using RNase P (Thermo)**

| RNase P Assay (250 reactions) | Thermo | 4485715 | 250 µL | $ 477.75 | $ 95.55 |
| --- | --- | --- | --- | --- | --- |
| **Total per 100 samples using** RNase P **as control:** | | | | | **$ 727.00** |

- **Internal Control – using B2M (custom designed primers)**

| B2M Forward Primer | IDT | N/A | 100 µM in 1027 µL | $ 5.89 | $ 0.02 |
| --- | --- | --- | --- | --- | --- |
| B2M Reverse Primer | IDT | N/A | 100 µM in 1042 µL | $ 6.83 | $ 0.02 |
| B2M Probe | IDT | N/A | 10 µM in 1020 µL | $ 274.04 | $ 0.81 |
| **Total per 100 samples using** B2M **as control:** | | | | | **$ 632.29** |

**Sample Processing**

**PCR Amplification**

**= $1173.91 CAD**

**= $1079.20 CAD**

**= $1026.50 CAD**

**= $931.79 CAD**

**Supplementary Figure 2**: Decision tree and total costs of saliva screening for 100 samples, excluding equipment cost. The cost for sample processing is shown as the start-up cost to buy the reagents needed to make the buffers for the protocol.
